# Exploring the impacts of learning modality changes: Validation of the learning modality change community of inquiry and self-efficacy scales

**DOI:** 10.1007/s10639-022-11258-3

**Published:** 2022-08-05

**Authors:** Yuane Jia, Peggy Gesing, Hyun-Jin Jun, Amanda K. Burbage, Thuha Hoang, Violet Kulo, Christina Cestone, Sarah McBrien, Joni Tornwall

**Affiliations:** 1 School of Health Professions, Rutgers, The State University of New Jersey, Newark, NJ USA; 2 School of Health Professions, Eastern Virginia Medical School, Norfolk, VA USA; 3 Health Professions Education, University of Maryland Baltimore, Baltimore, MD USA; 4 Department of Physical Therapy, Louisiana State University Health Sciences Center, New Orleans, LA USA; 5 College of Allied Health Professions, University of Nebraska Medical Center, Omaha, NE USA; 6 College of Nursing, The Ohio State University, Columbus, OH USA

**Keywords:** Cognition, Community of inquiry, Distance education, Factor analysis, Online learning, Self-efficacy

## Abstract

The rapid learning environment transition initiated by the COVID-19 pandemic impacted students’ perception of, comfort with, and self-efficacy in the online learning environment. Garrison’s Community of Inquiry framework provides a lens for examining students’ online learning experiences through three interdependent elements: social presence, cognitive presence, and teaching presence. Researchers in this study developed and validated the Learning Modality Change Community of Inquiry and Self-Efficacy scales to measure health professions students’ self-efficacy with online learning, while exploring how cognitive, social, and teaching presence is experienced by students who transition from one learning environment to another. The two scales demonstrate strong validity and reliability evidence and can be used by educators to explore the impacts of learning modality changes on student learning experiences. As learning environments continue to evolve, understanding the impact of these transitions can inform how educators consider curriculum design and learning environment changes.

## Introduction

Prior to the Coronavirus pandemic, much of medical and health professions education was delivered face-to-face with limited use of online teaching and learning methodologies (Enoch & Williams, 2021). The pandemic forced higher education institutions to transition quickly from face-to-face to remote teaching to comply with pandemic protocols (Almarzooq et al., 2020; Seah et al., 2021), resulting in challenges for all educational institutions. The challenges were exceptionally critical for health professions educators (HPEs) as students were prohibited from participating in labs and clinical field placements (Stokes, 2020).

Even under ideal conditions, online courses require careful planning and design, and effective online education requires a variety of instructional strategies to allow students to interact meaningfully with content, the instructor, and their classmates (Means et al., 2014). When educators responded to pandemic restrictions, they did not have time for careful design and development of online learning environments. Aspects of online learning, including technology policies and training, asynchronously-focused pedagogical decisions, robust assessment strategies, and quality assurance, were absent (Shisley, 2020). Instead, didactic learning was rapidly transitioned to a remote, synchronous environment, often referred to as online or blended (Schultz & DeMers, 2020), and clinical experiences transitioned to telemedicine, which led to complex barriers to learning for health professions students. As the pandemic progressed, faculty considered ways to transition from remote to deep online learning directed at students which included consideration for presence and engagement (Schultz & DeMers, 2020). To capture the range of experiences inclusive of remote learning, transition to deep online learning, and thoughtfully planned online learning, we use the term online learning.

Overall, students faced many challenges in the rapid transition to online learning that included obstacles to technology access, insufficient digital learning competencies, and difficulty navigating online academic honesty, privacy, and confidentiality (Turnbull et al., 2021). The move to online learning required students to establish new study routines without the structure and support of the campus setting (von Keyserlingk et al., 2022). These barriers and stressors required support to develop preparedness, access, and transferability between online learning and clinical skill performance (Shawaqfeh et al., 2020; Van Doren et al., 2020).

The rapid transition to online learning may have impacted student perceptions of their learning experience and online learning self-efficacy. Although researchers have explored self-efficacy in online learning environments, little research exists on the impact of transitions from face-to-face to online learning. This research project expands on the existing literature related to self-efficacy and student perception of meaningful learning in a collaborative environment through development and validation of a tool that measures health professions students’ self-efficacy with online learning while exploring how cognitive, social, and teaching presence is experienced by students who transition from one learning environment to another. Ultimately, students may experience social, teaching, and cognitive presence differently and have various levels of self-efficacy for online modalities as the learning environment was not chosen, but forced, due to the pandemic. Understanding the impact of learning environment transitions on student perceptions of their learning experience and self-efficacy can inform how educators approach online curriculum design and learning environments in health professions education.

### Health professions students and online learning

Most health professions education programs (e.g., medicine, physician assistant, and nursing programs) are offered primarily in a face-to-face environment; however, institutions are beginning to use online education platforms to expand the reach of their programs (Stuart & Triola, 2015; Taylor et al., 2019). These programs vary widely in structure and how technology supports their pedagogical approach (Cook et al., 2010). While some health professions programs have moved completely online or adopted hybrid approaches to education such as the flipped classroom model, other institutions have not embraced online learning as a modality of delivering instruction, particularly in the clinical context (Jones, 2015; Londgren et al., 2021).

Effective online teaching incorporates several basic concepts organized around types of interaction between the learner and the content, the teacher, and other learners (Means et al., 2014). Good teaching practices have been adapted for online instruction, often based on Chickering and Gamson’s (1999) principles of good practice, including encouraging interaction and higher-order thinking, providing opportunities for self-directed learning, formative and summative assessment, and effectively communicating task completion, high expectations, and diversity (Saiyad et al., 2020; Vyas et al., 2010). Adapting face-to-face instruction to an online learning environment requires faculty competencies in teaching and learning that represent social, pedagogical, managerial, and technical skills (Grant & Thornton, 2007; Saiyad et al., 2020; Tekian & Harris, 2012).

Transitioning to online learning in health professions education has been met with some resistance. Faculty report lack of technical skills, time, and institutional infrastructures that inhibit online learning development (Dyrbye et al., 2009; Niebuhr et al., 2014, Perlman et al., 2014). Poor student motivation, high anxiety, and poor interaction between learners and facilitators can hinder online learning, leading to concerns about the depth and breadth of learning and group communication skills (Regmi & Jones, 2020). Although each of these challenges has a solution rooted in communication, collaboration, and culture (O’Doherty et al., 2018), the pandemic forced educators to confront the issue with little to no time to implement these foundational solutions.

Despite hesitations about online health professions education, the outcomes of effective online learning are well documented. When comparing online learning experiences with traditional face-to-face experiences, researchers have not found significant differences in learning outcomes and student satisfaction (George et al., 2014). Moreover, when comparing interactive online learning environments that use discussion forums and other learning technologies to passive approaches, the interactive approach was found to improve knowledge and skills and student satisfaction (George et al., 2014). Although the rapid transition to online learning during the pandemic was challenging, it also provided an opportunity for educators to use what they already knew about online education to enhance their teaching with new online instructional strategies and skills to integrate well-planned online learning opportunities in their curriculum.

### Community of inquiry and self-efficacy

The Community of Inquiry (CoI) theoretical framework provides a lens for examining meaningful learning experiences through three interdependent elements – social presence, cognitive presence, and teaching presence (Garrison et al., 1999). Cognitive presence occurs with a triggering event or exploration in which information is exchanged and ideas are connected or applied. Social presence is the ability of participants in the learning environment to present themselves as real through emotional expression, open communication, and collaboration. Finally, teaching presence is understood as a function of course facilitation and course design which includes the “selection, organization, and primary presentation of course content, as well as the design and development of learning activities and assessment” (Garrison et al., 1999, p. 90). There are notable relationships between the constructs, with teaching presence demonstrating significant prediction of both cognitive and social presence (Garrison et al., 2001; Gutiérrez-Santiuste et al., 2015; Stenbom, 2018).

Self-efficacy considers students’ beliefs about what they can do with the skills and abilities that they have (Bong & Skaalvik, 2003) and refers to their beliefs in their capabilities to design and implement a course of action that leads to goal attainment (Bandura, 1977). Self-efficacy then influences choice of action, amount of effort, and length of perseverance in the face of obstacles. A strong sense of self-efficacy can lead to greater confidence in the ability to take on difficult tasks and challenges like online learning in order to develop skills (Alqurashi, 2016). Persistence in activities that may seem threatening but are safe can lead to mastery and enhancement of self-efficacy (Bandura, 1977). Students who have a strong sense of self-efficacy in their capacity to achieve tasks are motivated to take actions that make success more likely.

Much of the research on self-efficacy in online learning environments has been conducted in higher education but has focused on computer and internet self-efficacy (Alqurashi, 2016). However, connections have been made between computer self-efficacy and student satisfaction (Lee & Hwang, 2007; Lim, 2001) and intent to take online courses in the future (Lim, 2001). In addition, students with high self-efficacy in internet usage for information seeking exhibited higher self-efficacy for online learning (Tang & Tseng, 2013). Student readiness to learn online impacted satisfaction and is correlated with perceived effectiveness of e-learning (Almuwais et al., 2021). Students who persisted academically likely developed enhanced self-efficacy in the online learning environment. Self-efficacy motivates learner choice to initiate and persist with self-regulation and is essential to explain successful learner behaviors (Bandura, 1977; Bong & Skaalvik, 2003). Therefore, self-efficacy is a potentially important factor in an online learning environment where learner agency substitutes for traditional classroom structure (Shea & Bidjerano, 2010).

Connections have been made between the Community of Inquiry framework and learner self-efficacy with teaching presence positively predicting self-efficacy, and self-efficacy mediating the effect between social and cognitive presence (Lin et al., 2015). We used this study to further explore the connections between self-efficacy and the CoI framework by developing and validating a tool that measures self-efficacy with online learning while exploring how cognitive, social, and teaching presence is experienced by students who transition from one learning environment to another.

## Method

An interinstitutional team of health professions education (HPE) researchers from six U.S. universities and academic health centers developed a survey tool to investigate the impact of the rapid transition from face-to-face to online learning on students’ self-efficacy with online learning, attitudes toward online learning, and the factors impacting learning throughout the pandemic. The survey included 31 items on a six-point Likert scale from ‘strongly disagree’ (1) to ‘strongly agree’ (6). Each item asked health professions students to rate their agreement with statements about their experiences with online instruction and learning during the pandemic. The survey items were developed and reviewed by a group of researchers in health professions programs. The measurement constructs (self-efficacy, attitudes towards online learning, teaching presence, social presence, and cognitive presence) were developed from a thorough literature review. Based on a comprehensive literature review, important constructs and corresponding items related to student online learning were synthesized and revised to fit for measuring modality change purposes. The research team then ranked the items based on the necessity of each item and construct. Debriefing discussions and iterative revisions were made to ensure content and face validity prior to achieving consensus on the final 14 CoI and 13 self-efficacy items.

### Study procedures

Researchers distributed the student survey in fall 2021 to deans and health professions program directors at their respective institutions. Recruitment emails asked deans and directors to share the survey via email with students enrolled in health professions programs at their institutions from fall 2019 to fall 2021. The recruitment email contained a link to a Qualtrics online consent form which was followed by the 31 survey items. A series of demographic questions, including gender, race, age, field of study, and prior online learning experiences preceded the 31 survey items statements. The study was deemed exempt by the Institutional Review Boards at participating institutions where data collection and analysis required human subjects research review.

### Participants

A sample of 205 students from 5 institutions participated in the study. The sample consisted of students from a wide range of programs with the majority enrolled in Nursing (18.5%), Doctor of Medicine (MD) (17.6%), and Physician Assistant (12.2%) programs. Demographic data showed that 74.6% of participants were female and approximately 82% were under 35 years old. Seventy five percent of participants had online course experience prior to the pandemic; however, 64% indicated that they had “some” or “minimal experience” with online learning while only 22% indicated “quite a bit” or “a great deal” of experience. Demographic data are presented in Table 1.

**Table 1:** Demographic data

		n	%
Degree	Bachelors	38	18.54
	Masters	67	32.68
	Doctoral	93	45.37
	Certificates	6	2.93
Field of Study*	Nursing	38	18.50
	Doctor of Medicine (MD)	36	17.56
	Physician Assistant	25	12.20
	Health Sciences	14	6.80
	Other	25	12.20
Age	18–24 years old	76	37.07
	25–34 years old	92	44.88
	35–44 years old	22	10.73
	45 + years old	15	7.40
Gender	Male	48	23.41
	Female	153	74.63
	Other	4	2.00
Race	White	131	63.90
	Black	21	10.20
	Hispanic	13	6.30
	Asian	20	11.70
	Other	15	7.30
Online course exp before pandemic Y/N	Yes	153	74.63
	No	52	25.37
Level of online experience before the pandemic	None at all	29	14.10
	Minimal	61	29.80
	Some	71	34.6
	Quite a bit	24	11.70
	A great deal	20	9.80
Direct patient care during pandemic	0	108	52.68
	0–25%	47	22.93
	26–50%	19	9.27
	> 50%	30	14.60

* 22 different fields of study represented

### Measures

#### Community of inquiry

The Community of Inquiry (CoI) framework has been widely used to assess learners’ perceptions in the online and distance educational environments (Arbaugh et al., 2008; Garrison & Kanuka, 2004; Vaughan et al., 2013). The original CoI Survey includes 34 items based on the three interrelated components for successful learning: cognitive presence (12 items), social presence (9 items), and teaching presence (13 items). The reliability and validity of the original CoI survey and framework are well established in the literature (Stenbom, 2018), and high correlations have been found among the three components in previous research with various samples of students (Arbaugh et al., 2008; Bangert, 2009; Díaz et al., 2010; Kozan & Richardson, 2014; Shea et al., 2012; Stenbom, 2018; Swan et al., 2008). In the present study, the original CoI survey items were revised to create a new instrument with 14 items (4 cognitive presence, 5 social presence, and 5 teaching presence items) most relevant to the purpose of the study. The CoI items were adapted from the original CoI scale items and a Q-methodology study conducted by Ramlo (2021). Ramlo distilled 36 statements from a variety of sources including social media, student-led newspapers, and *The Chronicle of Higher Education*, and categorized them within seven themes related to the rapid, pandemic-related transition from face-to-face to online classes. The ideas represented in these statements filled in gaps where the student experience during the transition was not adequately reflected by the original CoI scale. The research team members, who have extensive knowledge in health professions and online education, modified the survey items to reflect the changes in the perceived impacts of online learning with traditional face-to-face classes. A common stem introduced the items for the CoI portion of the survey, which read as follows; "After my courses went online due to the pandemic..."

#### Self-efficacy

Self-efficacy, based primarily on Social Cognitive Theory (SCT), indicates one’s personal beliefs and perceived capacity to perform an action or behavior to complete a task successfully (Bandura, 1977). To assess perceived self-efficacy, 12 items were adopted from two existing self-efficacy scales. The 31-item Online Learning Self-Efficacy Survey (OLSS) is a reliable and valid tool to measure students’ preparedness, concerns, and learning needs for online learning as reflected by their self-efficacy, with an overall reliability of 0.95 (Sun & Rogers, 2021). Ten items from the OLSS were adapted for the present study (3 items from online learning task self-efficacy, 3 items from instructor and peer interaction and communication self-efficacy, and 4 items from self-regulation and motivation efficacy). Two additional items from Aguilera-Hermida’s (2020) self-efficacy scale were revised and added to the survey to cover aspects of self-efficacy not adequately covered by the OLSS: confidence in ability to be successful in online classes and to discuss topics with classmates and/or professors. One additional item was developed by the research team: confidence in learning new materials to achieve course objectives. A common stem introduced survey items gauging student self-efficacy after a learning modality change: “After experiencing a change in course delivery/learning modality as a result of the COVID-19 pandemic...”

### Statistical analysis

As a preliminary step, data were screened for missing data and univariate outliers across each item using IBM SPSS software, Version 28, RRID:SCR_016479. There were no significant outliers, and missing data ranged from 0.5% to 6.8% across items. Skewness and kurtosis were within acceptable range for all items (skewness ranging from –1.63 to 0.82; kurtosis < 3.21). Cronbach’s alpha reliability coefficients were estimated for each subscale of the new Learning Modality Change Community of Inquiry (CoI) and Self-Efficacy scales in SPSS. The Learning Modality Change CoI and Self-Efficacy scales were validated separately as two independent scales to create two instruments of reasonable length and provide flexibility for future researchers to choose to use one scale or both scales in a single survey. To assess the validity of the Learning Modality Change CoI and Self-Efficacy scales, exploratory structural equation modeling (ESEM) was performed using Mplus, Version 7.3, RRID:SCR_015578 with maximum likelihood estimation with robust standard errors (MLR) because this approach is robust to non-normal and missing data (Muthén & Muthén, 1998–2012). ESEM integrates the advantages of exploratory and confirmatory factor analysis and structural equation modeling by overcoming poor item-level factor structure fit and discriminant validity as well as biased structural parameter estimates (Marsh et al., 2009, 2014). ESEM provides “confirmatory tests of a priori factor structures, relations between latent factors and multigroup/multi-occasion tests of full (mean structure) measurement invariance” (Marsh et al., 2014, p. 85).

ESEM is known to be unbiased and comparable with confirmatory factor analysis in producing factor loadings and correlations without specifying the factor loading pattern (Asparouhov & Muthén, 2009; Sass & Schmitt, 2010; Schmitt, 2011). A goemin rotated solution was used for all models by allowing the correlations between the factors. On the basis of the recommendation of Kline (2016) and Hu and Bentler (1999), goodness of fit was assessed by multiple fit indices: chi-square (χ^2^) goodness-of-fit index, the comparative fit index (CFI) and the Tucker–Lewis index (TLI) ≥ 0.95 and 0.90. indicating excellent and acceptable fit; the root mean square error of approximation (RMSEA) and standardized root mean square residual (SRMR) ≤ 0.08, indicating a reasonable fit. Relative fit of different models was also considered, and a more parsimonious model is supported if there is a change in CFI of less than 0.01 (Chen, 2007) or a change in RMSEA of less than 0.015 (Chen, 2007). Additionally, factor loadings less than 0.4 (Stevens, 1992) and cross-loadings (Tabachnick & Fidell, 2001) were used in determining which items to drop from each revised scale.

## Results

Several ESEM models were compared to find the best fitting model for the Learning Modality Change CoI and Self-Efficacy scales. Model fit indices comparisons are presented in Table 2. The ESEM of three correlated factors of the Learning Modality Change CoI scale with 14 items achieved an acceptable model fit (χ2(52) = 116.98, *p* < 0.001, CFI = 0.96, TLI = 0.93, RMSEA = 0.08, SRMR = 0.02). Two of the items (11 and 14) had significant cross-loadings on Factors 2 and 3. However, Item 14 loaded much higher on Factor 3 (> 0.6) than on Factor 2 (< 0.4), which was not a concern. Since Item 11 had relatively closer cross-loadings at two factors, another ESEM analysis was performed after removing Item 11, resulting in a poorer model fit than the initial model with 14 items. We retained Item 11 in Factor 3 because that item addresses motivation related to online learning, which is an important element to consider in measurement of cognitive presence. Although the initial model with 14 items had cross-loadings on Item 11, retaining it on Factor 3 was deemed acceptable because it demonstrated a higher loading there (> 0.4) than on the other Factor (< 0.4).

**Table 2:** ESEM fit indices with MLR estimator

	Chi-square/df	CFI^1^	TLI^2^	RMSEA^3^	SRMR^4^
Self-efficacy model with 13 items	130.162/42	0.937	0.883	0.101	0.027
Self-efficacy model removing item 5	77.027/33	0.965	0.929	0.081	0.024
Self-efficacy model removing item 1	54.573/33	0.982	0.964	0.056	0.02
Self-efficacy model without item 1 and 5	47.996/25	0.979	0.954	0.067	0.019
CoI model with 13 items	116.977/52	0.961	0.931	0.08	0.024
CoI model removing item 11	111.724/42	0.953	0.913	0.093	0.024

1. comparative fit index; 2. Tucker–Lewis Index; 3. root mean squared error of approximation; 4. standardized root m

A similar analysis procedure was applied to the Learning Modality Change Self-Efficacy scale. The ESEM of the three correlated factors in the 13-item self-efficacy scale failed to achieve a good model fit as evidenced by the RMSEA and TLI not falling within the recommended limits of the fit indices (χ2(42) = 130.16, *p* < 0.001, CFI = 0.94, TLI = 0.88, RMSEA = 0.10, SRMR = 0.03). Item 1, I feel confident in my ability to be successful in online classes, and Item 5, I am able to learn new material to achieve course objectives, showed significant and moderate cross-loadings on Factors 1 and 3. After removing Item 1, model fit significantly improved (χ2(33) = 54.57, *p* < 0.001, RMSEA = 0.06, CFI = 0.98, TLI = 0.96, SRMR = 0.02); however, Item 5 had a factor loading smaller than 0.3 at the planned Factor 1 but a significant factor loading at Factor 3. Alternatively, after removing Item 5, the model produced an acceptable model fit, but Item 2 had very close and significant cross-loadings at Factor 2 and Factor 3. After looking at the fit indices and factor loadings as well as reexamining the meaning of the factor, Items 1 and 5 were removed from the model, yielding a favorable final model with 11 items (χ2(25) = 48.00, *p* < 0.001, CFI = 0.98, TLI = 0.95, RMSEA = 0.07, SRMR = 0.02). No significant and close cross-loadings were present in the final model.

Standardized factor loadings and factor correlations for the final ESEM of the Learning Modality Change CoI and Self-Efficacy scales in the student sample are presented in Tables 3 and 4, respectively. The internal consistency (Cronbach’s alpha) of the Learning Modality Change CoI and Self-Efficacy scales were 0.92 and 0.95, respectively. The internal reliability for each of the three subscales of Learning Modality Change CoI scale ranged from 0.89 to 0.92. The internal reliability for each of the three subscales of the Learning Modality Change Self-Efficacy scale ranged 0.78 to 0.92. Factor correlations for the self-efficacy scale ranged from 0.45 to 0.66. Factor correlations for the CoI scale ranged from 0.59 to 0.71. Additionally, all subscales of the Learning Modality Change CoI scale were found to be positively associated with subscales of Learning Modality Change Self-Efficacy scale, with correlations ranging from 0.34 to 0.72, all p values < 0.001, and evidence of good concurrent validity.

**Table 3:** ESEM solution: three factors based on 14 community of inquiry items

#	Items	F1 (Teaching)	F2 (Social)	F3 (Cognitive)
1	The instructors were able to guide the class effectively to completing the course activities*	**0.884**	-0.172	0.053
2	It was equally as easy (or easier) for me to communicate with my instructors as it was when the classes were face-to-face**	**0.542**	0.161	0.126
3	The instructors provided feedback to me online in an equally timely fashion as it was provided to me in face-to-face classes*	**0.838**	-0.002	-0.057
4	I had the sense that the instructors were present in the course and attentive to students' needs to the same degree that they were before courses moved online*	**1.072**	-0.255	-0.004
5	My instructors seemed to teach more effectively in the online environment than they did in the face-to-face environment*	**0.627**	0.048	0.231
6	I felt more comfortable participating in online discussions with peers than I did when my course was face-to-face*	0.005	**0.572**	0.246
7	I was able to collaborate with peers in the online classroom more effectively than I was in the face-to-face classroom*	-0.051	**0.691**	0.258
8	There was a sense of collegial trust among my peers in the online environment that was greater than it was in the face-to-face classroom*	-0.006	**0.704**	0.165
9	I was equally involved in interactions with peers as I was in face-to-face courses**	0.006	**0.966**	-0.154
10	I was equally involved in interactions with my instructors as I was in face-to-face courses**	0.06	**0.84**	-0.001
11	I was more motivated to engage in learning activities in the online version of my courses than I was in the face-to-face version of the courses before the pandemic*	0.106	**0.364**	**0.476**
12	I developed a greater appreciation for online learning through participation in online classes after the pandemic began than I had before the pandemic**	0.012	0.089	**0.602**
13	I learned more in the online version of the course than I would have in the face-to-face version of the course**	0.135	-0.002	**0.802**
14	The learning activities in the online course were more engaging than the face-to-face learning activities*	-0.002	**0.342**	**0.61**
**Factor Correlation**				
F1		1	0.71	0.588
F2			1	0.69
F3				1
**Cronbach’s alpha**		0.9	0.92	0.89

Bold loadings were significant, *p* < 0.05

*Items from Swan et al., CoI scale. Teaching Presence α = 0.94, Social Presence α = 0.91, Cognitive Presence α = 0.95

**Items from Ramlo, S. Reliability score not reported

**Table 4:** ESEM solution: three factors based on 11 self-efficacy items

#	Items	F1 (Task Self-Efficacy)	F2 (Interaction Self-Efficacy)	F3 (Self-Regulation efficacy)
1	I feel confident in taking an online quiz/test*	**0.466**	-0.001	**0.298**
2	I feel confident in viewing my online course materials in the Learning Management System (e.g., BlackBoard)*	**0.775**	-0.019	0.002
3	I feel confident in submitting course assignments through the Learning Management System (e.g., BlackBoard)*	**0.953**	0.002	-0.229
4	I feel confident in my ability to discuss topics with classmates and/or professors in an online course***	**0.213**	**0.568**	0.081
5	I can develop a sense of community through interactions with other online course participants*	0	**0.991**	-0.094
6	I can develop a sense of community through interactions with my online instructors*	0.002	**0.937**	-0.016
7	I can develop a sense of collaboration through teamwork/group projects in my online courses*	0.078	**0.814**	0
8	I can motivate myself to persist in my online courses when facing difficulties or setbacks*	0.002	0.143	**0.79**
9	I can motivate myself to explore content-related questions in my online courses*	-0.07	0.223	**0.744**
10	I can manage study time for my online courses by setting goals*	0.023	-0.127	**0.857**
11	I can encourage myself to understand the most difficult materials presented in an online course*	-0.012	0.005	**0.898**
**Factor Correlation**				
F1		1	0.452	0.656
F2			1	0.608
F3				1
**Cronbach’s alpha**		0.78	0.92	0.91

Bold loadings were significant, *p* < 0.05

*Items from Sun, Y., & Rogers, R. Online Learning Self-efficacy Scale (OLSS). Overall reliability with 31 items = 0.95

**Item from Aguilera-Hermida, A. P., Reliability score not reported

## Discussion

### Results summary

The ESEM confirmed three factors for the final Learning Modality Change CoI scale: cognitive presence with 4 items, social presence with 5 items, and teaching presence with 5 items. All items loaded as we originally conceptualized on their respective factors with all loadings greater than 0.4 (Table 3). Additionally, consistent with Lin et al. (2015), all subscales of the Learning Modality Change CoI scale were found to be positively associated with self-efficacy subscales. This supports results from a study by Shea and Bidjerano (2010) who found that teaching presence and social presence were significantly correlated with student self-efficacy. Compared to the original 34 item CoI survey (Abbitt & Boone, 2021; Arbaugh et al., 2008), the new 14-item instrument developed in the present study provides strong validity and reliability in measurement of cognitive, social, and teaching presence after changes in learning modality in health professions education programs. While CoI scales have been used to measure the experiences of students studying in a pre-existing online environment, this research lends support for the use of the CoI framework to study effects of learning modality changes on health professions students’ learning experiences.

The ESEM confirmed three factors for the final Learning Modality Change Self-Efficacy scale: online learning task self-efficacy with 3 items, interaction and communication self-efficacy with 4 items, and self-regulation and motivation with 4 items. All 11 items loaded as we originally conceptualized, with all loadings greater than 0.45 as presented in Table 4. Items aligned well with the original subscales of the OLSS (Sun & Rogers, 2021). The new, shorter version of the scale that resulted from this study demonstrated favorable construct validity and internal reliability in measuring health professions students’ online learning self-efficacy after a shift in learning modalities.

### Strengths and limitations

Overall, the two new instruments Learning Modality Change CoI and Self-Efficacy scales produced in this study demonstrated substantial validity and reliability. These scales provide a reliable, shorter, and more efficient measurement of the CoI and self-efficacy of students who experience a change in learning modality. The Learning Modality Change CoI Scale can be used to measure the impacts of learning modality changes on students’ perceptions of cognitive, social, and teaching presence. The Online Learning Self-Efficacy Scale can be used to measure changes in self-efficacy of students transitioning from one learning modality to another. The two scales can be used separately or together, depending on the research context and survey requirements. The wide range of health professions programs and institutions represented by the student sample in this study supports the external validity of the findings. The new scales are efficient yet comprehensive measures of the constructs they represent and are ready to be used by researchers in health professions education. In addition, these scales can be used separately or together in other educational fields with adjustments to survey stems and further validation.

Moreover, the application of ESEM technique showed a promising approach to validate a modified measurement tool. As evidenced by moderate to high correlation among the three factors of CoI, especially social presence with teaching and cognitive presence, there is potential overlap between the three factors. Not surprisingly, social presence is more difficult to achieve in an online environment than in a face-to-face setting, and measurement of the social presence construct typically overlaps with teaching and cognitive presence (Garrison et al., 2001; Gutiérrez-Santiuste et al., 2015; Stenbom, 2018). Further work with another student sample and in other educational contexts might help clarify the issue. Future work might include a larger sample of students to conduct measurement invariance analyses across groups such as gender, race, field of study, or online experience.

### Implications and conclusion

It is important to differentiate between carefully planned online learning that occurs under ideal conditions and the rapid transition to remote learning that many students experienced at the start of the COVID-19 pandemic. Online course environments require extensive planning and design prior to the start of the course, including considerations for cognitive, social, and teaching presence (Garrison et al., 1999). Online learning calls for a variety of teaching and technology tools that lead to meaningful interactions with content, instructors, and classmates (Means et al., 2014). The rapid transition to online learning during the pandemic did not allow educators to carefully plan their online learning environments, nor did it account for instructors’ and students’ lack of comfort and self-efficacy with online learning environments and tools. Much of the instruction that occurred was conducted via remote, synchronous learning environments where instructors attempted to replicate their face-to-face teaching practices amid challenges with access to technology, students experienced increased temptation to engage in academic dishonesty, and students and instructors expressed concern about privacy and confidentiality, (Turnbull et al., 2021).

Although faculty and students faced challenges in the transition, the move to online learning environments in higher education provided opportunities for institutions to reconsider their curricular designs and approaches to delivery of instruction. Higher education is under ongoing pressure to meet the dynamic needs of society, and the pandemic made a significant impact on current and future educational practices (Mbhiza, 2021). The rapid transition to online learning ultimately led to a paradigm shift where online learning environments gained value as student and faculty comfort evolved. Online learning widens access to education for student populations historically underrepresented in higher education including those from low socioeconomic backgrounds, students with disabilities, regional and remote students, indigenous students, and first-generation students (Stone, 2017). The increased value and comfort with online learning provides an opportunity for health professions education programs who are tasked with increasing diversity in the health occupations workforce (Bouye et al., 2016; Jackson & Gracia, 2014) to increase access to underrepresented student populations (Gumport, 2016; Letizia, 2017). Increasing access to health professions education through expansion of online learning opportunities demands an understanding of the impact of transitions in learning modalities on student self-efficacy and learning experiences and outcomes.

This study expands on the existing literature about self-efficacy with online learning and the Community of Inquiry Framework through development of the Learning Modality Change CoI and Self-Efficacy Scales. As higher education institutions evaluate future use of online learning environments, this study provides a tool for examining the impacts of transitions to online learning on student self-efficacy for learning and cognitive, social, and teaching factors that influence their learning experience and outcomes. While this tool was developed for health professions education, it can be used to measure changes in all student populations as they transition from one learning environment to another.

## Declarations

The datasets generated during and/or analyzed during the current study are available from the corresponding author on reasonable request.

## References

[CR1] Abbitt JT, Boone WJ (2021). Gaining insight from survey data: An analysis of the community of inquiry survey using Rasch measurement techniques. Journal of Computing in Higher Education.

[CR2] Aguilera-Hermida AP (2020). College students’ use and acceptance of emergency online learning due to covid-19. International Journal of Educational Research Open.

[CR3] Almarzooq ZI, Lopes M, Kochar A (2020). Virtual learning during the covid-19 pandemic: A disruptive technology in graduate medical education. Journal of the American College of Cardiology.

[CR4] Almuwais A, Alqabbani S, Benajiba N, Almoayad F (2021). An emergency shift to e-learning in health professions education: A comparative study of perspectives between students and instructors. International Journal of Learning, Teaching and Educational Research.

[CR5] Alqurashi E (2016). Self-efficacy in online learning environments: A literature review. Contemporary Issues in Education Research.

[CR6] Arbaugh JB, Cleveland-Innes M, Diaz SR, Garrison DR, Ice P, Richardson JC, Swan KP (2008). Developing a community of inquiry instrument: Testing a measure of the community of inquiry framework using a multi-institutional sample. The Internet and Higher Education.

[CR7] Asparouhov T, Muthén B (2009). Exploratory structural equation modeling. Structural Equation Modeling: A Multidisciplinary Journal.

[CR8] Bandura A (1977). Self-efficacy: Toward a unifying theory of behavioral change. Psychological Review.

[CR9] Bangert AW (2009). Building a validity argument for the community of inquiry survey instrument. The Internet and Higher Education.

[CR10] Bong M, Skaalvik EM (2003). Academic self-concept and self-efficacy: How different are they really?. Educational Psychology Review.

[CR11] Bouye KE, McCleary KJ, Williams KB (2016). Increasing diversity in the health professions: Reflections on student pipeline programs. Journal of Healthcare, Science and the Humanities.

[CR12] Chen FF (2007). Sensitivity of goodness of fit indexes to lack of measurement invariance. Structural Equation Modeling: A Multidisciplinary Journal.

[CR13] Chickering AW, Gamson ZF (1999). Development and adaptations of the seven principles for good practice in undergraduate education. New Directions for Teaching and Learning.

[CR14] Cook DA, Garside S, Levinson AJ, Dupras DM, Montori VM (2010). What do we mean by web–based learning? A systematic review of the variability of interventions. Medical Education.

[CR15] Díaz SR, Swan K, Ice P, Kupczynski L (2010). Student ratings of the importance of survey items, multiplicative factor analysis, and the validity of the community of inquiry survey. The Internet and Higher Education.

[CR16] Dyrbye L, Cumyn A, Day H, Heflin M (2009). A qualitative study of physicians' experiences with online learning in a masters degree program: Benefits, challenges, and proposed solutions. Medical Teacher.

[CR17] Enoch TR, Williams RC (2021). Why face-to-face medical education will prevail despite the world’s swift acclimatisation to virtual learning. Postgraduate Medical Journal.

[CR18] Garrison DR, Anderson T, Archer W (1999). Critical inquiry in a text-based environment: Computer conferencing in higher education. The Internet and Higher Education.

[CR19] Garrison DR, Anderson T, Archer W (2001). Critical thinking, cognitive presence, and computer conferencing in distance education. American Journal of Distance Education.

[CR20] Garrison DR, Kanuka H (2004). Blended learning: Uncovering its transformative potential in higher education. The Internet and Higher Education.

[CR21] George PP, Papachristou N, Belisario JM, Wang W, Wark PA, Cotic Z, Rasmussen K, Sluiter R, Riboli-Sasco E, Tudor Car L, Masulanov EM, Molina JA, Heng BH, Zhang Y, Wheeler EL, Shorbaji NA, Majeed A, Car J (2014). Online eLearning for undergraduates in health professions: A systematic review of the impact on knowledge, skills, attitudes and satisfaction. Journal of Global Health.

[CR22] Grant MR, Thornton HR (2007). Best practices in undergraduate adult-centered online learning: Mechanisms for course design and delivery. Journal of Online Learning and Teaching.

[CR23] Gumport PJ, Bastedo MN, Altbach PG, Gumport PJ (2016). Graduate education and research: Interdependence and strain. American higher education in the 21st century: Social, political, and economic challenges.

[CR24] Gutiérrez-Santiuste E, Rodríguez-Sabiote C, Gallego-Arrufat MJ (2015). Cognitive presence through social and teaching presence in communities of inquiry: A correlational–predictive study. Australasian Journal of Educational Technology.

[CR25] Hu L, Bentler PM (1999). Cutoff criteria for fit indexes in covariance structure analysis: Conventional criteria versus new alternatives. Structural Equation Modeling: A Multidisciplinary Journal.

[CR26] Jackson CS, Gracia JN (2014). Addressing health and health-care disparities: The role of a diverse workforce and the social determinants of health. Public Health Reports.

[CR27] Jones SH (2015). Benefits and challenges of online education for clinical social work: Three examples. Clinical Social Work Journal.

[CR28] Kline RB (2016). Principles and practice of structural equation modeling.

[CR29] Kozan K, Richardson JC (2014). New exploratory and confirmatory factor analysis insights into the community of inquiry survey. The Internet and Higher Education.

[CR30] Lee, J.-K., & Hwang, C.-Y. (2007). The effects of computer self-efficacy and learning management system quality on e-Learner’s satisfaction. In L. Cameron, A. Voerman, & J. Dalziel (Eds.), *Proceedings of the 2007 European LAMS Conference: Designing the future of learning* (pp. 73–79). LAMS Foundation.

[CR31] Letizia AJ (2017). Using strategic planning to create the public good for higher education in volatile times. International Journal of Progressive Education.

[CR32] Lim CK (2001). Computer self-efficacy, academic self-concept, and other predictors of satisfaction and future participation of adult distance learners. American Journal of Distance Education.

[CR33] Lin S, Hung TC, Lee CT (2015). Revalidate forms of presence in training effectiveness: Mediating effect of self-efficacy. Journal of Educational Computing Research.

[CR34] Londgren MF, Baillie S, Roberts JN, Sonea IM (2021). A survey to establish the extent of flipped classroom use prior to clinical skills laboratory teaching and determine potential benefits, challenges, and possibilities. Journal of Veterinary Medical Education.

[CR35] Marsh HW, Morin AJ, Parker PD, Kaur G (2014). Exploratory structural equation modeling: An integration of the best features of exploratory and confirmatory factor analysis. Annual Review of Clinical Psychology.

[CR36] Marsh HW, Muthén B, Asparouhov T, Lüdtke O, Robitzsch A, Morin AJS, Trautwein U (2009). Exploratory structural equation modeling, integrating CFA and EFA: Application to students' evaluations of university teaching. Structural Equation Modeling: A Multidisciplinary Journal.

[CR37] Mbhiza HW (2021). Shifting paradigms: Rethinking education during and post-COVID-19 pandemic. Research in Social Sciences and Technology.

[CR38] Means B, Bakia M, Murphy R (2014). Learning online: What research tells us about whether, when and how.

[CR39] Muthén, L. K., & Muthén, B. O. (1998–2012). *Mplus user's guide* (7th ed.). Muthén & Muthén.

[CR40] Niebuhr V, Niebuhr B, Trumble J, Urbani MJ (2014). Online faculty development for creating E-learning materials. Education for Health.

[CR41] O’Doherty D, Dromey M, Lougheed J, Hannigan A, Last J, McGrath D (2018). Barriers and solutions to online learning in medical education–an integrative review. BMC Medical Education.

[CR42] Perlman R, Christner J, Ross P, Lypson M (2014). A successful faculty development program for implementing a sociocultural ePortfolio assessment tool. Academic Medicine.

[CR43] Ramlo S (2021). COVID-19 response: Student views about emergency remote instruction. College Teaching.

[CR44] Regmi K, Jones L (2020). A systematic review of the factors – enablers and barriers – affecting e-learning in health sciences education. BMC Medical Education.

[CR45] Saiyad S, Virk A, Mahajan R, Singh T (2020). Online teaching in medical training: Establishing good online teaching practices from cumulative experience. International Journal of Applied & Basic Medical Research.

[CR46] Sass DA, Schmitt TA (2010). A comparative investigation of rotation criteria within exploratory factor analysis. Multivariate Behavioral Research.

[CR47] Schmitt TA (2011). Current methodological considerations in exploratory and confirmatory factor analysis. Journal of Psychoeducational Assessment.

[CR48] Schultz RB, DeMers MN (2020). Transitioning from emergency remote learning to deep online learning experiences in geography education. Journal of Geography.

[CR49] Seah, B., Ang, E. N. K., Liaw, S. Y., Lau, S. T., & Wang, W. (2021). Curriculum changes for pre-registration nursing education in times of covid-19: For the better or worse? *Nurse Education Today*, *98*. 10.1016/j.nedt.2020.104743 10.1016/j.nedt.2020.104743 PMC7771900 33421745

[CR50] Shawaqfeh MS, Al Bekairy AM, Al-Azayzih A, Alkatheri AA, Qandil AM, Obaidat AA, Harbi SA, Muflih SM (2020). Pharmacy students perceptions of their distance online learning experience during the covid-19 pandemic: A cross-sectional survey study. Journal of Medical Education and Curricular Development.

[CR51] Shea P, Bidjerano T (2010). Learning presence: Towards a theory of self-efficacy, self-regulation, and the development of a communities of inquiry in online and blended learning environments. Computers & Education.

[CR52] Shea P, Hayes S, Smith SU, Vickers J, Bidjerano T, Pickett A, Gozza-Cohen M, Wilde J, Jian S (2012). Learning presence: Additional research on a new conceptual element within the Community of Inquiry (CoI) framework. The Internet and Higher Education.

[CR53] Shisley, S. (2020). Emergency remote learning compared to online learning. *Learning Solutions*, 1–15. https://learningsolutionsmag.com/articles/emergencyremote-learning-compared-to-online-learning

[CR54] Stenbom S (2018). A systematic review of the Community of Inquiry survey. The Internet and Higher Education.

[CR55] Stevens JP (1992). Applied multivariate statistics for the social sciences.

[CR56] Stokes DC (2020). Senior medical students in the covid-19 response: An opportunity to be proactive. Academic Emergency Medicine.

[CR57] Stone, C. (2017). *Opportunity through online learning: Improving student access, participation and success in higher education*. National Center for Student Equity in Higher Education. https://apo.org.au/node/94591.

[CR58] Stuart, G., & Triola, M. (2015). Enhancing health professions education through technology: building a continuously learning health system. In *Proceedings of a Conference Recommendations* (pp. 9–12).

[CR59] Sun Y, Rogers R (2021). Development and validation of the Online Learning Self-efficacy Scale (OLSS): A structural equation modeling approach. American Journal of Distance Education.

[CR60] Swan, K., Richardson, J. C., Ice, P., Garrison, D. R., Cleveland-Innes, M., & Arbaugh, J. B. (2008). Validating a measurement tool of presence in online communities of inquiry. *E-Mentor, 2*(24). https://www.e-mentor.edu.pl/artykul/index/numer/24/id/543

[CR61] Tabachnick BG, Fidell LS (2001). Using multivariate statistics.

[CR62] Tang Y, Tseng H (2013). Distance learners’ self-efficacy and information literacy skills. Journal of Academic Librarianship.

[CR63] Taylor S, Iacobelli F, Luedke T, Matthews PA, Monge M, Cooper J, Moreira J, Grippo P, Girotti J, Molina Y, Yanez B, Simon MA (2019). Improving health care career pipeline programs for underrepresented students: Program design that makes a difference. Progress in Community Health Partnerships: Research, Education, and Action.

[CR64] Tekian A, Harris I (2012). Preparing health professions education leaders worldwide: A description of masters-level programs. Medical Teacher.

[CR65] Turnbull D, Chugh R, Luck J (2021). Transitioning to e-learning during the covid-19 pandemic: How have higher education institutions responded to the challenge?. Education and Information Technologies.

[CR66] Van Doren EJ, Lee JE, Breitman LS, Chutinan S, Ohyama H (2020). Students’ perceptions on dental education in the wake of the covid-19 pandemic. Journal of Dental Education.

[CR67] Vaughan ND, Cleveland-Innes M, Garrison DR (2013). Teaching in blended learning environments: Creating and sustaining communities of inquiry.

[CR68] von Keyserlingk L, Yamaguchi-Pedroza K, Arum R, Eccles JS (2022). Stress of university students before and after campus closure in response to covid-19. Journal of Community Psychology.

[CR69] Vyas R, Anshu, Lata H, Burdick W, Singh T (2010). Application of classroom good teaching practices to an online faculty development programme in India. South-East Asian Journal of Medical Education.

